# Thiamine Deficiency in Adolescents with Eating Disorders: A Prospective Cohort Study

**DOI:** 10.3390/nu12051396

**Published:** 2020-05-13

**Authors:** Hilla Bahat, Gad Reisler, Nurit Brandriss, Adina Bar-Chaim, Michael Goldman

**Affiliations:** 1 Department of Pediatrics, Shamir Medical Center, Zerifin 70300, Israel; gold@shamir.gov.il; 2 Sackler School of Medicine, Tel Aviv University, Tel Aviv 69978, Israel; reisler@shamir.gov.il (G.R.); NuritBr@shamir.gov.il (N.B.); adinab@shamir.gov.il (A.B.-C.); 3 Outpatient Day Hospital for Eating Disorders, Shamir Medical Center, Zerifin 70300, Israel; 4 Clinical Chemistry Laboratory, Shamir Medical Center, Zerifin 70300, Israel

**Keywords:** pediatric eating disorder, thiamine deficiency, dietary intervention

## Abstract

Background: Pediatric eating disorders (PED) patients are prone to nutritional deficiencies. Thiamine deficiency is well described in other malnutrition states but is not routinely screened for in PED. In the current study we evaluated the prevalence of thiamine deficiency among PED patients on their first admission to an outpatient day hospital for eating disorders (DH). Methods: In this prospective cohort study, we measured whole blood thiamine pyrophosphate concentrations (TPP) in addition to a routine laboratory workup in 69 girls on their first admission to DH. Two subgroup analyses were performed: (I) Patients with a previous dietary intervention (“diet” group, *n* = 30) or naïve-to-treatment patients (“naïve” group, *n* = 39) and (II) Type of PED: Restrictive (group R, *n* = 44) or binge-eating/purging (group BP, *n* = 25). Results: Thiamine deficiency was identified in four girls (6%), all in the “naïve” group. Three of them had BP, and one had R. Patients in the “diet” group had a significantly higher TPP compared to the “naïve” group (55.5 µg/L vs. 46.7 µg/L, *p* = 0.004). TPP levels returned to normal after two weeks of the treatment program in all deficient patients. Conclusion: Thiamine deficiency was uncommon among PED patients and was easily replenished. Screening for deficiency should be performed among treatment-naïve patients. Keynotes: Whole blood thiamine pyrophosphate concentrations (TPP) are seldom screened for among PED patients. In the current study, we detected thiamine deficiency in only 6% of patients on their first admission to an outpatient day hospital for eating disorders. All deficient patients did not have a recent dietary intervention. We recommend considering screening for thiamine deficiency in treatment-naïve PED patients.

## 1. Introduction

Patients with eating disorders (ED) suffer from multiple medical complications, mostly linked to weight loss, malnutrition and purging [1]. Vitamin and mineral deficiencies are described among these patients [2,3], but the prevalence of micronutrient deficiencies and their consequences in malnourished patients with ED are poorly known. Indeed, few studies have focused on patients with moderate undernutrition and the results are occasionally conflicting [4].

Thiamine is an important cofactor in biochemical pathways in many systems, and its deficiency may result in neuropsychiatric morbidity [5]. The time to deplete thiamine body stores is 4–6 weeks [6]; there are well known predisposing factors for thiamine deficiency, like malnutrition, alcohol abuse, bariatric surgery and hyperemesis gravidarum [5,6].

Whole blood thiamine pyrophosphate concentrations (TPP) are not routinely screened for in ED patients, and the literature regarding thiamine deficiency, especially in pediatric ED (PED) patients, is sparse [7,8,9,10,11]. In a 2010 review [12], it remains unclear whether thiamine deficiency is indeed an issue for concern among ED patients.

The current study aimed to screen and follow all PED patients admitted for the first time to the outpatient day hospital for eating disorders in Shamir Medical Center (DH) for thiamine deficiency. We hypothesize that thiamine deficiency is under-recognized in PED patients, and would be detected even among unsuspected cases.

## 2. Materials and Methods

In this prospective cohort study, we evaluated TPP and other clinical and laboratory features among 11–17-year-old girls with ED, at the time of their first admission to our treatment program in DH, between 2013–2014. All subjects gave their written informed consent for inclusion before they participated in the study.

### 2.1. Study Participants

The diagnosis of ED was based upon the American Psychiatric Association’s Diagnostic and Statistical Manual of Mental Disorders, Fifth Edition (DSM-5) [13], as was the subtype diagnosis. For the statistical analysis, due to the small number of patients, we divided our patients into two groups according to restrictive (R) or binge-eating/purging (BP) only.

Patients referred to DH from the community when they required a more intensive treatment setting, or when interventions in the community failed due to lack of compliance or severity of illness. Thus, at the time of their first evaluation in our institution, some patients already initiated a prior intervention at the community, including a balanced diet and a multivitamin supplement (with thiamine 1.5 mg—125 percent daily values). We screened all patients regarding a previous intervention in the past month on admission. Our patient cohort was thus divided into two groups–patients with a previous intervention before admission (“diet” group) and naïve-to-treatment patients (“naïve” group). Of note, we do not know the specifics of the prior interventions performed in the community among the “diet” group, and we assume they were not homogenous.

### 2.2. Methods

The routine parameters that were collected on admission in addition to TPP include:

Age, percent of ideal body weight (% IBW)—the 50th percentile of body mass index (BMI) for age, type of ED, previous ambulatory intervention (reported by the patients or documented in the referral papers), documented neurological examination, use of psychiatric medications and routine protocol blood tests (including vitamin B12 levels, albumin and hemoglobin).

Whole blood thiamine pyrophosphate concentration (TPP) was determined using a commercial kit: Chromsystems Instruments and Chemicals GmbH, Munich, Germany, CE, IVD (in vitro diagnostic) in high-performance liquid chromatography (HPLC), equipped with a fluorescence detector (Ex367nm, Em435nm), available online: https://www.chromsystems.com/vitamin-b1-in-whole-blood-hplc-35000.html (accessed on 10 May 2020) [14]. In this method of analysis, the reference range of the thiamine pyrophosphate (TPP), in whole blood, is 28–85 µg/L. All samples were tested using the same kit. Thiamine deficiency was defined as whole blood thiamine concentration <28 µg/L.

In girls with documented thiamine deficiency, TPP were repeated 2 weeks into the treatment program.

The study was approved by the Institutional Research Ethics Committee of Shamir Medical Center. Trial registration: ClinicalTrials.gov Identifier: NCT02164487, March 2013.

### 2.3. Treatment Program

The treatment program in DH included nutritional rehabilitation, medical stabilization, psychiatric evaluation, individual and group therapy, schooling and family intervention.

Nutritional rehabilitation consisted of a balanced diet tailored to patient requirements and preferences. Some patients required the addition of an enteral formula. Patients not receiving an enteral formula were administered a multivitamin that included thiamine (1.5 mg—125 percent daily values) and calcium. All patients admitted to DH went through a similar treatment program.

### 2.4. Data Analysis

Sample size calculation was based on a previous study on whole blood thiamine concentration in 50 healthy French volunteers that found a standard deviation (SD) of 15 µg/L [15]. Hence, seventeen patients in each group were required in order to detect a 15 µg/L difference in mean whole blood thiamine concentration with a power of 80% and alpha of 0.05. In order to overcome a possibly higher SD and to allow for subgroup analysis, 69 patients were recruited.

Categorical variables were compared by χ2 or Fisher exact tests, and continuous variables were compared using the Mann–Whitney U test. The difference between the subgroups was tested using univariate analysis of variance (two-way ANOVA). Statistical analysis was performed with SPSS 24 (IBM) statistics Version 24.

## 3. Results

The study group included 69 girls with PED, 44 girls (64%) with R and 25 (36%) with BP. Thirty patients (43%) had a previous intervention in the month prior to admission (“diet” group) and 39 (57%) were naïve-to-treatment. Neurological examination was normal in all patients and there were no other specific clinical findings suggesting thiamine deficiency. Fifteen girls were treated with psychiatric medications (selective serotonin reuptake inhibitor/ atypical antipsychotics) prior to their admission, fourteen of them in the “diet” group.

R patients were younger than BP patients (14.8 vs. 15.7 years, *p* = 0.02) and had a lower%IBW (87.7 vs. 107.6, *p* < 0.001).

Thiamine deficiency was diagnosed in 4 girls (6%), all were naïve-to-treatment. Three had BP and 1 had R, a difference that did not reach statistical significance (*p* = 0.132). TPP was retested in the deficient patients after 2 weeks of treatment in our program and returned to normal in all four patients.

The comparison between patients with or without a previous intervention is shown in Table 1. The comparison between patients according to type of ED is shown in Table 2.

TPP was higher among patients in the “diet” group (55.5 µg/L vs. 46.7 µg/L, *p* = 0.004), but was not influenced by the type of PED (49.8 µg/L in R, 51.8 µg/L in BP, *p* = 0.3).

In a univariate analysis of variance (two-way ANOVA), there was a significant effect of a previous intervention on TPP (*p* = 0.001), but the effect of ED type on TPP was statistically insignificant (*p* = 0.7).

None of the patients had B12 deficiency, anemia or low albumin levels. 

## 4. Discussion

To our knowledge, this study is the largest published screening specifically focused on thiamine deficiency among PED patients. There are a few previous case reports describing pediatric patients with ED and severe neurological manifestations such as Wernicke’s encephalopathy (WE) that have led to the diagnosis of thiamine deficiency [7,8,9]. In a recent systematic review by Oudman et al., 12 adult and pediatric cases of WE in AN were described. The authors speculated that since the symptoms of AN and WE may overlap, the diagnosis is often delayed [10]. Winston et al. screened adult patients with AN for thiamine deficiency, and found that 38% were indeed deficient, without relation to vomiting, alcohol consumption or duration of eating restraint [11].

In the current study, we checked TPP in 69 girls that were referred for treatment of PED in our hospital. Surprisingly, thiamine deficiency was documented only in four patients (6%). These findings may be explained by more awareness, and earlier treatment of ED among children compared to adult patients, as reflected by the high rate of previous dietary interventions among our cohort.

Moreover, none of the patients with thiamine deficiency had a recent dietary intervention; PED patients with a recent dietary intervention had higher TPP than those who were treatment-naive (55.5 µg/L vs. 46.7 µg/L, *p* = 0.004)); and the deficiency was rapidly corrected after two weeks of general nutritional rehabilitation, that included a multivitamin containing thiamine (1.5 mg—125 percent daily values) or enteral formula, without using a high-dose thiamine supplement.

Malnutrition secondary to eating disorders usually develops slowly and homeostasis of micronutrients is often maintained for long periods of time. Hence, much so, that normal micronutrient laboratory results among ED patients are common and can often be misinterpreted as a normal nutritional state [12]. Indeed, even large studies of micronutrient deficiency among severely malnourished ED patients only detected specific deficiencies in about 50% of the patients [4,16]. This can perhaps explain the low percentage of thiamin deficiency detected in our patient cohort, coupled with the ease of replenishment in the deficient girls.

Our study has a few limitations. First, we do not know the specifics of the community interventions performed before admission to our institution, and they were probably heterogeneous interventions among the different patients. Still, it seems, that no matter what the interventions were, all patients with previous treatment were non-deficient. Furthermore, since thiamine deficiency was uncommon among our study cohort in general, our sample size was too small to detect the significance of eating disorder type on thiamine deficiency.

## 5. Conclusions

In contrast to our study hypothesis, thiamine deficiency was uncommon among PED patients. Moreover, it was easily replenished following a short intervention in all deficient cases. Interestingly, we only detected the deficiency among naïve patients, who were not treated thus far. Since the deficiency was asymptomatic in all our cases, but can still have a detrimental effect, we recommend considering screening for thiamine deficiency in all naïve-to-treatment PED patients.

## Figures and Tables

**Table 1:** Comparison between eating disorder (ED) patients, with or without prior treatment.

	Prior Treatment (“diet”) 30/69 (43%)	Naïve-to-Treatment 39/69 (56%)	*p*-Value
Thiamine pyrophosphate µg/L mean [± standard deviation (SD)]	55.5 (10.6)	46.7 (12.5)	0.004
Age years mean (± SD)	15.1 (1.6)	15.2 (1.3)	0.91
% of ideal body weight (± SD)	98.5 (18.3)	92.2 (11.4)	0.16

* Mann–Whitney U test.

**Table 2:** Comparison between patients with restrictive (R) and binge-eating/purging (BP) disorder.

	R 44/69 (64%)	BP 25/69 (36%)	*p*-Value
Thiamine pyrophosphate µg/L mean (± SD)	49.8 (11)	51.8 (14.8)	0.3
Age years mean (± SD)	14.8 (1.5)	15.7 (1.2)	0.02
% of ideal body weight (± SD)	87.7 (9.4)	107.6 (14.7	<0.001

* Mann–Whitney U test.
